# Cutaneous infection by *Mycobacterium haemophilum *and *kansasii *in an IgA-deficient man

**DOI:** 10.1186/1471-5945-11-3

**Published:** 2011-01-26

**Authors:** Vassiliki Bekou, Amanda Büchau, Michael J Flaig, Thomas Ruzicka, Michael Hogardt

**Affiliations:** 1 Department of Dermatology and Allergology, Ludwig-Maximilians-University, Munich, Germany; 2 Max von Pettenkofer-Institut für Hygiene and Medical Microbiology, Ludwig-Maximilians-University, Munich, Germany; 3 Bavarian Health and Food Safety Authority, Department of Infectiology, Oberschleissheim, Germany

## Abstract

**Background:**

The prevalence of infections by nontuberculous mycobacteria (NTM) has steadily increased over the past decades, especially in immunocompromised patients.

**Case presentation:**

We present a patient with IgA-deficiency and mixed cutaneous infection by two slowly growing mycobacteria, *Mycobacterium *(*M.*) *haemophilum *and *M. kansasii.*

**Conclusions:**

Cutaneous *M. haemophilum *infections most often result from HIV or transplantation-associated immunosuppression. Rarely, *M. haemophilum *may also infect healthy patients or iatrogenically immunosuppressed patients without transplantation. *M. kansasii *is one of the most frequent NTM and large awareness exists about its involvement in human diseases. Mycobacterial diagnosis of cutaneous infections should be considered in long-lasting skin lesions.

## Background

Immunoglobulin (Ig) A-deficiency is the most common primary antibody deficiency. Although the majority of affected individuals have no apparent symptoms, selected patients suffer from recurrent mucosal infections, allergies, and autoimmune diseases [1]. So far, in patients with selective IgA deficiency no infections with nontuberculous mycobacteria (NTM) have been reported. However, in the last decade NTM are increasingly recognized as infective agents, particularly among immunocompromised patients.

*Mycobacterium haemophilum *is an established cause of cutaneous infections in immunocompromised hosts [2]. The most common clinical manifestation of *M. haemophilum *infections are skin lesions with a preference for cooler body sites such as extremeties, while the development of sporotrichoid-like nodular lymphangitis is exceptional.

*M. kansasii *is one of the most frequent NTM causing human diseases among both immunocompetent and immunocompromised patients [3]. *M. kansasii *most likely causes infections resembling tuberculosis likewise with a preference for pulmonary disease [4]. Cutaneous infections due to this slow growing mycobacterium are rare and may resemble cellulitis or sporotrichosis. Nevertheless, *M*. *kansasii *should be included in the differential diagnosis of skin infections with an indolent course and lack of response to standard antibiotics [5]. Since NTM are typically ubiquitous in the natural environment several routes of transmission may be considered [3]. Unfortunately, the environmental source of *M. haemophilum *is still unknown, while tap water is likely the major reservoir for *M. kansasii *infections. Inoculation of NTM into the host usually occurs due to impairment of the physical skin barrier. Affected patients commonly show a history of fish tank cleaning, oyster shucking, swimming, or other aquatic activities [6].

## Case presentation

**Figure 1:**
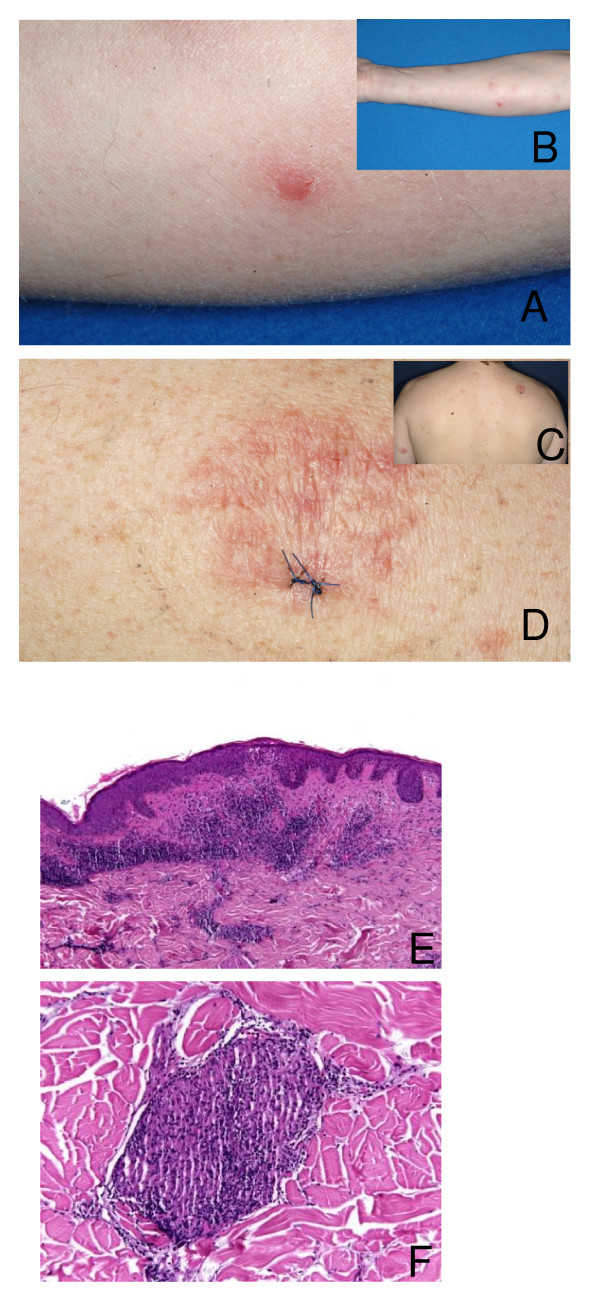
**Clinical and histological findings**. On the right arm (A) erythematous nodules (B) were found in a sporotrichoid-like manner. At the back (C) a sharply marginated erythematous plaque (D) was seen. The histopathology of the skin revealed in the dermis disseminated caseating partly epitheloid cell granulomas with a marginal zone of lymphocytes and a subepidermal lymphohistiocytic infiltrate with perivascular accentuation (E). Close up of an epitheloid cell granuloma (F).

A 48-year old man presented with multifocal nodules of variable size arranged in a sporotrichoid-like manner on the right arm, the dorsum of the digit IV, the left arm, the right side of his back, the knees and a partly circumscribed erythematous plaque on his back (Figure 1A-D). The lesions persisted for already 2 months without any other symptoms.

At two occasions skin biopsies from several lesions (dorsum of right digit IV, dorsum of the right hand, the right elbow, and the patients back) were initiated and subjected in parallel to histopathological and microbiological examinations. They revealed caseating epitheloid cell granuloma in the dermis with a marginal zone of lymphocytes and a subepidermal lymphohistiocytic infiltrate with perivascular accentuation (Figure 1E, F). By PAS-, Fite-, Ziehl-Neelsen and Auramin staining no acid fast bacilli were detected. *Borrelia*-specific IgM and IgG and tuberkulin skin test were negative. Microbiological cultures showed twice growth of *M. haemophilum *from independent lesions on the right hand taken about 8 weeks apart. Interestingly, *M. kansasii *was recovered from another lesion on the right elbow indicating a mixed infection.

*Mycobacteria*-specific liquid and solid cultures were performed by using the automated BACTEC MGIT 960 system (Becton Dickinson) at 36°C and Lowenstein-Jensen Agar (LJA) at 24°C, 30°C, and 36°C. Growth of acid fast bacilli was detected from LJA at 30°C supplemented with hemin discs. First, culture isolates were identified as NTM due to the absence of an amplicon by a *M. tuberculosis*-specific *gyrB *PCR [7] and were then further verified by the GenoType Mycobacterium DNA strip technology (Hain Lifescience, Nehren, Germany), namely the GenoType Mycobacterium CM and AS assay (CM, common mycobacteria; AS, additional species) [8]. The GenoType Mycobacterium CM assay enables the simultaneous identification of several species, including those of the *M*. *tuberculosis *complex, the *M*. *avium *complex, *M*. *abscessus*, *M*. *kansasii *and *M*. *chelonae *etc. The Mycobacterium AS test provides the identification of rarely found species, such as *M*. *simiae*, *M*. *haemophilum*, and *M. mucogenicum*. In case of *M*. *haemophilum*, Mycobacterium CM assay revealed a banding pattern specific for the *M*. *haemophilum*-*M*. *nebraskense*-*M*. *malmoense*-*M*. *palustre *group. Further species differentiation was done with the AS assay that showed positivity for *M*. *haemophilum *and *M*. *nebraskense *but not for *M*. *malmoense *or *M*. *palustre*. The growth requirement for hemin indicated the presence of *M. haemophilum. *Nevertheless, species identification of *M. haemophilum *was confirmed at the National Reference Center for Mycobacteria in Borstel (Germany). *M*. *kansasii *was recovered with the Bactec MGIT 960 and identified with the GenoType Mycobacterium CM assay.

Thus, culture positive lesions were all from the right body site, while no co-infection of a single lesion was found. Interestingly, the patient was working as a scaffolder having potential exposure to diverse environmental habitats while touring around Europe. Further, he is aquarist but reports to usually wear gloves during cleaning his aquarium.

Interestingly, in the patient's history a selective IgA-deficiency was documented since childhood. His mother also suffered from IgA-deficiency. Since infancy human immunoglobulin (Beriglobin^®^) was administered, but due to his frequent travel activities the substitution was realized irregularly. Serum IgA level was 1.8 (07-4.0) mg/ml under substitution with Beriglobin^®^. Biochemical laboratory parameters were within normal ranges except for serum protein electrophoresis with albumin of 57.2% (59-70.6) and beta globulin of 14.9% (7.3-12.2). ASL, AST, ACE, ANA, and ENA tests were unremarkable. Besides multiple cutaneous lesions there was no evidence for lymphadenopathy or systemic infection with *M. haemophilum/M. kansasii *by using abdominal ultrasound, thorax X-ray, and thoraco-abdominal computer tomography imaging. Mycobacterial sputum cultures were negative as well. Empirical triple treatment with clarithromycine 2 × 500 mg/d, rifabutine 1 × 300 mg/d and ethambutol 1 × 20 mg/kg body weight/d was initiated. This regime was given for 6 months, while a complete clinical remission of skin lesions was seen within 4 months. Finally, two years after his initial presentation the patient is still healthy.

## Discussion

NTM can be categorized by the growth rate in slowly growing and rapidly growing species, pigmentation (pigmented and non-pigmented species), and optimal growth temperature, which can provide helpful information for a preliminary classification of NTM. *M. marinum, M. kansasii*, and *M. avium-intracellulare *are examples of slow-growing mycobacteria. *M. fortuitum, M. chelonea, and M. abscessus *are examples of rapidly growing mycobacteria [1,2]. Diagnosis relies on skin biopsy, histopathologic examination, microbiological culture and molecular detection of mycobacterial DNA.

Strikingly, cutaneous *M. haemophilum *infections are most often the result of HIV- or transplantation-associated immunosuppression. It's most common clinical manifestations are cutaneous lesions with a preference for cooler body sites such as extremities. Therefore, in case of skin biopsies LJA at 30°C should routinely supplemented with hemin discs in order to fulfill special growth requirements of *M. haemophilum *that is known to primarily cause cutaneous infections. Rarely, *M. haemophilum *may infect iatrogenically immunosuppressed patients without transplantation or even healthy persons. The development of sporotrichoid nodular lymphangitis due to *M. haemophilum *is exceptional [3].

In contrast, *M. kansasii *infection has been reported in up to 20 percent of nontuberculous mycobacteriosis in both immunocompetent and immunocompromised patients. Disseminated infections are uncommon but of poor prognosis [9]. The outcome of *M. kansasii *pulmonary infection is good if diagnosed and treated early. In one report, *M. kansasii *cutaneous infection occurred on the hand and forearm, with carpal tunnel syndrome complicated by concomitant pulmonary *M. tuberculosis *[6], and in another as cellulitis in a patient with systemic lupus erythematosus [4].

As the only comorbid condition our patient showed IgA-deficiency. Sustained, very low levels of IgA, IgG, or IgM, as found in primary immunodeficiency syndromes, are associated with significantly increased risk for infections, primarily respiratory tract infections of bacterial origin. IgA, a major serum Ig and the predominant antibody in external secretions that bathe mucosal surfaces, plays a key role in immune protection. IgA at concentrations of about 2-3 mg/ml is the second most prevalent serum antibody after IgG, which is normally present at about 12 mg/ml. However, IgA-deficiency is the most common primary antibody deficiency and appears to be well tolerated. Due to the large surface area of the human mucosa of up to 400 m^2^, the role of IgA in the protection of this surface from harmful environmental agents is extremely essential. Any changes connected with a lack or an overproduction of IgA can manifest as variable clinical disease [10]. Most individuals with IgA-deficiency have no apparent symptoms, but selected patients suffer from recurrent mucosal infections, allergies, and autoimmune diseases [1]. Obviously, this biological diversity has both genetic and environmental components. For instance, it is known that IgA is secreted with sweat (sIgA) assuming an important immunological function on skin. Moreover, lower cutaneous sIgA levels and increased skin infections have been reported in patients with atopic dermatitis, while these patients of course often present also an impairment of the normal skin barrier. In conclusion, there are some clues about an association of skin infections with IgA-deficiency, although no clear pathophysiological link may be provided [11-13]. Strikingly, there is increasing evidence that serum IgA is able to trigger effector functions that have the potential to destroy microorganisms and mammalian cells. It has long been recognized that compared with IgM and IgG, IgA is a poor activator of complement. IgA does not activate the classical pathway and its role in activation of the alternative pathway remains controversial [14]. However, it may have a role in activation of phagocytic system by means of the FcRα receptors. It has been proposed that serum IgA binds to FcRα receptor on the monocytes and granulocytes; thereby, immune complexes formed by foreign antigens and IgA are cleared from the circulation by the phagocytic system without activating the complement system and without causing inflammation. Serum IgA may also have a role in controlling the immune system through inhibition of neutrophil chemotaxis by binding to other inhibitory proteins such as α-1-antitrypsin and forming complexes. Bacteria of the intestinal tract, oral cavity, and respiratory and genital tracts are coated with secretory IgA. As a result, the epithelial adherence and penetration of bacteria are limited, and the bacteria are confined to the mucosal surfaces [15]. Rodriguez et al. suggest that IgA may play a role in protection against mycobacterial infections in the respiratory tract by blocking the pathogen entrance and/or by modulating the pro-inflammatory responses [16].

Most likely, in our patient NTM's are transmitted from a contaminated environmental source by a direct cutaneous route. The exact source and the infection mode remain unclear. Nevertheless, even in a healthy individual an appropriate exposition (e.g. skin injury, "high" bacterial burden) may lead to NTM infections, as described for *M. marinum*. In IgA-deficiency, beside reduced serum IgA levels often also other immune deficiencies e.g. in cytokine levels and B-cell function but also associated T cell defects have been described. Thus, we cannot exclude that IgA-deficiency may be a surrogate for another undetected less marked immune dysfunction [12].

## Conclusions

This is the first report of a mixed cutaneous infection *M. haemophilum *and *M. kansasii*, both belonging to the slow growing mycobacteria, in a patient with IgA-deficiency. The patient is aquarist and was working as a scaffolder presenting a relevant risk of exposure to diverse environmental habitats while touring around Europe. Although according to current knowledge no definite pathophysiological link between mycobacterial disease and IgA-deficiency may be given, we cannot rule out that this immunological imbalance may contribute to the patient's vulnerability towards NTM. Finally, the patient responded well to triple anti-mycobacterial therapy with complete regression of entire skin lesions within 4 months.

## Consent

We obtained a written informed consent from the patient for publication of this article and accompanying images. A copy of the written consent is available for the review by the Editor-in-Chief of this journal.

## Competing interests

The authors declare that they have no competing interests.

## Authors' contributions

VB and MH conceived the project and wrote the manuscript. AB and TR helped in the acquisition of clinical data and manuscript revision. MF carried out and interpreted the histological examinations. All authors read and approved the final manuscript.

## Pre-publication history

The pre-publication history for this paper can be accessed here:

http://www.biomedcentral.com/1471-5945/11/3/prepub
